# Survival prediction in colorectal cancer liver metastases using machine learning with SHAP-based interpretation

**DOI:** 10.3389/fonc.2026.1836991

**Published:** 2026-06-10

**Authors:** Nan Li, Baoxin Dong, Yu Liang, Likun Liu, Xixing Wang, Ce Zhang, Shulan Hao

**Affiliations:** 1 Department of Oncology, Shanxi Provincial Research Institute of Traditional Chinese Medicine, Taiyuan, Shanxi, China; 2 Graduate School, Shanxi University of Traditional Chinese Medicine, Taiyuan, Shanxi, China; 3 Key Laboratory of Cellular Physiology, Ministry of Education, Department of Physiology, Shanxi Medical University, Taiyuan, Shanxi, China

**Keywords:** colorectal cancer, colorectal cancer liver metastasis, liver metastasis, machine learning, predictive models, prognostic prediction model, shapley additive explanations (SHAP), traditional Chinese medicine

## Abstract

**Background:**

Colorectal cancer liver metastasis (CRLM) remains a leading cause of cancer-related mortality worldwide. Accurate prognostic stratification is crucial for making individualized therapeutic decisions. Conventional statistical approaches are limited in capturing complex nonlinear interactions among multidimensional clinical variables. This study aimed to develop, temporally validate, and deploy an interpretable machine learning (ML) model incorporating Traditional Chinese Medicine (TCM) intervention to predict long-term survival in patients with CRLM.

**Methods:**

A retrospective cohort of 861 CRLM patients was included following institutional ethical approval. Clinical, pathological, and treatment-related variables, including TCM exposure characteristics, were systematically collected. After data preprocessing and feature selection, six machine learning algorithms—Random Forest (RF), XGBoost, K-Nearest Neighbors (KNN), Support Vector Machine (SVM), LightGBM, and CatBoost—were trained using five-fold cross-validation to predict 36- and 60-month overall survival. Model performance was evaluated using the area under the receiver operating characteristic curve (AUC), precision–recall curve (PRC), and confusion matrix metrics. The best-performing model was further validated in an temporal dataset to assess generalizability. Model interpretability was enhanced using SHapley Additive exPlanations (SHAP), and the final optimized model was deployed as a web-based clinical application to facilitate individualized survival prediction and real-time risk stratification.

**Results:**

Among candidate models, the optimized XGBoost algorithm demonstrated superior predictive performance. For 36-month survival prediction, the AUC reached 0.891 in the training cohort and 0.833 in the testing cohort, with consistent performance for 60-month survival prediction. Temporal validation confirmed model robustness and stability. SHAP analysis revealed that TNM stage, liver metastasis burden, and TCM intervention intensity were among the most influential prognostic factors. TCM exposure exhibited a protective association with survival probability in a dose-dependent pattern. The web-based tool enables clinicians to input individual patient parameters and obtain dynamic risk estimates with transparent, interpretable outputs.

**Conclusions:**

We developed and temporal validated an interpretable ML-based prognostic model for CRLM and successfully translated it into a web-based clinical decision-support tool. By integrating TCM intervention into predictive modeling, this study provides quantitative evidence supporting its potential survival benefit. The deployed model offers a practical and accessible instrument for personalized prognostic assessment and optimized treatment planning for CRLM patients.

## 1 Introduction

Colorectal cancer (CRC) is one of the most common malignancies of the digestive system. It ranks third in incidence among all cancers, accounting for approximately 10% of newly diagnosed cancer cases worldwide, and its incidence continues to rise. CRC also ranks second in cancer-related mortality, making it one of the leading causes of cancer death. According to the Global Cancer Statistics 2020, Asia accounted for 49.3% of newly diagnosed CRC cases and 58.3% of CRC-related deaths worldwide (1). Approximately 15% of patients present with liver metastasis at the time of diagnosis, and nearly 50% of CRC patients will develop liver metastasis during the course of their disease (2). Colorectal cancer liver metastasis (CRLM) is one of the leading causes of death among patients with CRC. Although early diagnosis and surgical treatment can significantly improve survival, most patients are already at the metastatic stage at the time of diagnosis, resulting in poor prognosis, with a five-year survival rate of less than 30% (3).

The prognosis of CRLM is influenced by multiple factors, which can generally be classified into clinical, biological, and molecular factors. Clinical factors include tumor burden, anatomical distribution, and the timing of metastasis. Biological factors, such as serum CEA levels and changes in inflammatory markers, have also been shown to be closely associated with the prognosis of CRLM. At the molecular level, alterations including RAS/BRAF MSI status and HER2 alterations may also affect patient outcomes (4). Several prognostic models have been developed to stratify patients with CRLM. Among them, the Fong Clinical Risk Score (CRS) is one of the most widely used clinical scoring systems, incorporating factors such as lymph node status of the primary tumor, disease-free interval, number and size of liver metastases, and preoperative CEA levels. However, this scoring system is primarily based on static clinicopathological variables and does not account for the impact of therapeutic interventions on patient outcomes (5).

Based on data extracted from the Surveillance, Epidemiology, and End Results (SEER) database for patients diagnosed with Tis–T2 stage colorectal cancer between 2010 and 2015, independent prognostic factors for liver metastasis included sex, race, primary tumor size, serum CEA levels, and chemotherapy status (6). In addition, a retrospective study demonstrated that tumor volume measured by CT imaging was associated with overall survival (OS) and recurrence-free survival (RFS) after initial resection of liver metastases (7). Among patients undergoing resection of liver metastases with fewer than 12 lymph nodes evaluated, a lymph node ratio greater than 0.22 was independently associated with poor prognosis and progression-free survival (PFS) (8).

Furthermore, Traditional Chinese Medicine (TCM) is characterized by a holistic regulatory pattern involving “multi-component, multi-target, and multi-pathway” synergistic effects, enabling multi-level intervention in tumor biological processes. Previous studies have shown that TCM combined with radiotherapy and chemotherapy can enhance antitumor efficacy in various solid tumors while reducing treatment-related adverse reactions and improving patient tolerance and treatment compliance (9). However, whether TCM intervention is also a prognostic factor influencing outcomes in patients with CRLM has not yet been investigated.

With advances in data science, machine learning (ML) has emerged as an increasingly important tool in cancer diagnosis and prognostic assessment. By integrating and analyzing large volumes of clinical, genomic, and imaging data, ML approaches can generate high-performance predictive models that enable clinicians to evaluate disease status more accurately and at earlier stages (10). A range of ML algorithms—including support vector machines (SVM), random forests, XGBoost, LightGBM, and CatBoost—have demonstrated strong predictive capabilities and considerable potential in early disease prediction, leading to their widespread adoption in oncological research. For example, ML models have been applied to predict recurrence in breast cancer (11). In studies of lung malignancies, ML techniques have been used to analyze pre-treatment FDG-PET/CT images to predict tumor progression and OS (12). Similarly, in patients with hepatocellular carcinoma, six ML-based prognostic models have been developed to successfully predict overall survival in individuals with positive AFP levels (13). Collectively, these findings highlight the growing value of machine learning approaches in the early detection and prognostic prediction of cancer.

In recent years, machine learning has been increasingly applied to prognostic prediction in CRLM. Previous studies have developed models integrating six key features: LDH, CA19−9, alanine ALT, CEA, TBIL, and the albumin-to-globulin ratio (14). However, these studies have largely overlooked the potential impact of TCM interventions on CRLM prognosis. Future research should therefore investigate how TCM-based interventions might be incorporated as predictive factors within existing prognostic models.

Although the strong predictive power of machine learning approaches is widely recognized, their decision-making processes are often difficult to interpret, rendering them “black boxes” and limiting their clinical applicability. To address this challenge, model interpretability methods have emerged in recent years. Among these, SHAP (SHapley Additive exPlanations)—a game theory–based interpretative tool—quantifies the contribution of each feature to model predictions, providing insight into the underlying decision-making process. In oncology prognostic models, SHAP can elucidate the specific impact of individual features, such as clinical variables, gene expression levels, or treatment regimens, on patient outcomes, thereby enhancing model transparency and trustworthiness (15).

In this study, we utilized a retrospective cohort dataset and incorporated TCM interventions to develop and validate an interpretable machine learning model aimed at quantifying key risk factors for CRLM. This model is intended to function as a web-based tool, providing prognostic support for clinicians and patients and thereby facilitating more individualized and precise therapeutic decision-making.

## 2 Materials and methods

### 2.1 Data source and study design

This retrospective study included patients with CRLM who were treated at the Oncology Department of Shanxi Provincial Hospital of Traditional Chinese Medicine between January 1, 2015, and December 31, 2023, with follow-up extending to November 2025. Inclusion criteria were (1) pathologically confirmed primary colorectal or colon cancer; and (2) the presence of synchronous or metachronous liver metastases. Exclusion criteria were (1) concurrent other primary malignancies, (2) patients with unknown survival time, and (3) cases with substantial missing data.

### 2.2 Ethical statement

This study was approved by the Institutional Review Board of Shanxi Provincial Research Institute of Traditional Chinese Medicine (approval number: 2025KY-08075; **Supplementary Figure 1**). Given the retrospective design and the use of de-identified patient data, the requirement for informed consent was waived. All methods were performed in accordance with the relevant guidelines and regulations.

### 2.3 Model development and comparison

The original dataset was first preprocessed by excluding variables with more than 30% missing values, including BMI (45.4%), differentiation (39.0%), gross type (57.9%), CEA (40.8%), and CA199 (42.1%). Missing data mainly resulted from the retrospective nature of the study, particularly because some outpatient medical records contained relatively simplified documentation, leading to incomplete recording of certain clinicopathological variables. For the remaining missing data, continuous variables were imputed using multiple imputation, while categorical variables were filled with the mode. Univariate Cox regression analysis was then performed to screen clinical and pathological features associated with CRLM, and variables with statistical significance (P < 0.05) were incorporated into a multivariate Cox regression model to identify independent prognostic factors.

For model development, the cohort was randomly split into training and testing sets at a 7:3 ratio. Six machine learning algorithms were evaluated: random forest (RF), XGBoost (version 2.0.3), k-nearest neighbors (KNN), SVM, LightGBM, and CatBoost. Hyperparameters for each model were optimized on the training set using five-fold cross-validation combined with grid search. Model performance was subsequently assessed on the testing set using receiver operating characteristic (ROC) curves, precision-recall (PRC) curves, and confusion matrices, allowing identification of the optimal model for predicting 3- and 5-year survival in CRLM patients.

To evaluate the efficacy of TCM, TCM was defined as oral administration of standardized Chinese herbal decoctions prescribed in routine clinical practice, typically taken as one prescription per day in two divided doses (morning and evening). TCM exposure was quantified based on the average annual dosage of herbal medicine and categorized into six ordinal levels for model development. Categorize For survival analysis, patients were further grouped into low-, medium-, and high-exposure categories to allow for more intuitive clinical interpretation, and Kaplan–Meier analysis was performed to assess treatment outcomes. Finally, the model was validated using an temporal dataset.

### 2.4 Model interpretability

As described in the background, SHAP is a technique for interpreting machine learning model predictions. Based on the concept of Shapley values from game theory, SHAP quantifies the contribution of each feature to the model’s output. A higher absolute SHAP value indicates a stronger influence of the feature on the prediction, while the sign of the SHAP value intuitively reflects whether the feature increases or decreases the predicted probability. This approach enables in-depth analysis of the decision-making logic of otherwise “black-box” models.

### 2.5 Web application

To enhance the clinical applicability and translational value of the predictive model, we developed an interactive web application using the R Shiny framework, which was deployed on the shinyapps.io platform. The application allows users to input basic demographic characteristics and relevant clinical variables, and it generates individualized risk predictions based on the optimal machine learning model developed in this study. To overcome the inherent “black-box” nature of machine learning models, the application integrates a SHAP-based interpretability module. By computing SHAP values, the module quantifies the marginal contribution of each input feature to the individual prediction, providing transparent and intuitive explanations of model decisions at the patient level.

### 2.6 Statistical analysis

All statistical analyses were performed using Python (version 3.12.2). Continuous variables are presented as medians with interquartile ranges and were assessed for normality using the Kolmogorov–Smirnov (K–S) test. Comparisons of continuous variables were conducted using the Mann–Whitney U test or the Kruskal–Wallis H test, as appropriate. Categorical variables are expressed as counts and percentages and were compared using the chi-square test or Fisher’s exact test. Survival analyses were performed using the Kaplan–Meier method to estimate overall survival, and differences between groups were assessed with the log-rank (Mantel–Cox) test. Survival time is reported as median survival with 95% confidence intervals (95% CI). A two-sided p-value < 0.05 was considered statistically significant.

## 3 Results

### 3.1 Clinical characteristics of patients with CRLM

**Figure 1:**
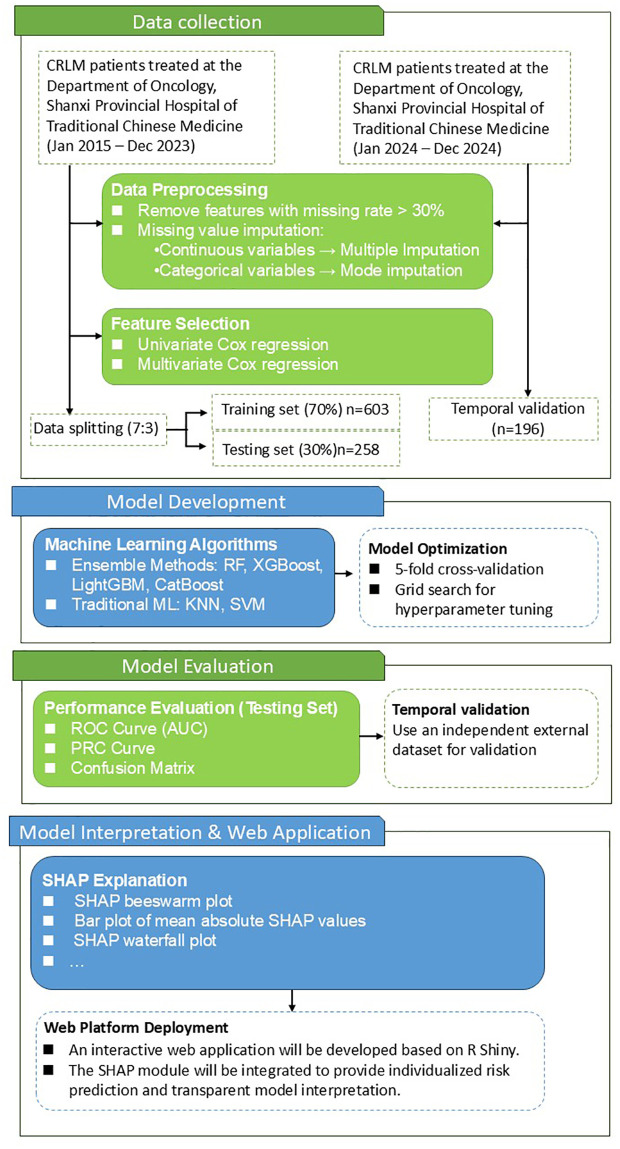
Technical workflow of the machine learning–based prognostic model for patients with CRLM. The workflow includes data collection, data preprocessing, feature selection, model development using multiple machine learning algorithms, model evaluation and temporal validation, followed by model interpretation using SHAP and deployment of an interactive web application based on R Shiny for individualized risk prediction and transparent model explanation.

The overall study design is illustrated in **Figure 1**. A total of 861 eligible patients with CRLM were included. After imputation of missing values, the clinical and pathological characteristics of the cohort are summarized in **Table 1**.

**Table 1:** Baseline characteristics of patients with colorectal cancer liver metastasis.

Characteristic		Cases (%)	Characteristic		Cases (%)
Age	<40	34(3.95)	Sex	Female	358(41.58)
	40-65	495(57.49)		Male	503(58.42)
	>65	332(38.56)	Hypertension	Negative	593(68.87)
TCM Doses	<90	616(71.54)		Positive	268(31.13)
	90-180	132(15.33)	Diabetes	Negative	740(85.95)
	180-270	53(6.16)		Positive	121(14.05)
	270-360	22(2.56)	Coronary_Heart_Disease	Negative	806(93.61)
	360-450	15(1.74)		Positive	55(6.39)
	>450	23(2.67)	Stroke	Negative	819(95.12)
Primary Site	Rectum	368(42.74)		Positive	42(4.88)
	Colon	132(15.33)	Hepatitis_B	Negative	852(98.95)
	Transverse colon	20(2.32)		Positive	9(1.05)
	Rectosigmoid junction	15(1.74)	Extrahepatic Metastasis	Negative	391(45.41)
	Double primary tumors	4(0.46)		Positive	470(54.59)
	Left hemicolon	185(21.49)	Liver Resection	Negative	758(88.04)
	Right hemicolon	137(15.91)		Positive	103(11.96)
TNM Stage	I	5(0.58)	Chemotherapy	Negative	67(7.78)
	II	54(6.27)		Positive	794(92.22)
	III	147(17.07)	Liver Radiotherapy	Negative	830(96.4)
	IV	655(76.07)		Positive	31(3.6)
Histology Primary	Adenocarcinoma	802(93.15)	Liver Intervention	Negative	708(82.23)
	Squamous cell carcinoma	5(0.58)		Positive	153(17.77)
	Mucinous adenocarcinoma	32(3.72)	Liver HIFU	Negative	859(99.77)
	Neuroendocrine carcinoma	13(1.51)		Positive	2(0.23)
	Intraepithelial neoplasia	5(0.58)	Immunotherapy	Negative	791(91.87)
	Signet-ring cell carcinoma	4(0.46)		Positive	70(8.13)
Primary-to-liver metastasis interval (months)	0	514(59.7)	Targeted Therapy	Negative	409(47.5)
	<3	52(6.04)		Positive	452(52.5)
	4-6	44(5.11)	Neoadjuvant Chemotherapy	Negative	776(90.13)
	7-12	92(10.69)		Positive	85(9.87)
	13-36	115(13.36)	Status	Alive	58(6.74)
	>36	44(5.11)		Death	803(93.26)
Number of Liver Metastases	Single	48(5.57)			
	2-3	36(4.18)			
	4-5	10(1.16)			
	Multiple	767(89.08)			

More than half of the patients were diagnosed between 40 and 65 years of age, while 39% (n = 630) were older than 65 years. Male patients predominated, accounting for 58% of the cohort. The vast majority of primary tumors were adenocarcinomas (93%). Synchronous liver metastases were observed in 60.0% of patients, 89.0% of whom presented with multiple hepatic lesions. Overall, 55% of patients had metastases at other sites. Only 12% underwent hepatic resection, whereas 92% received chemotherapy. Immunotherapy was administered to 8% of patients, targeted therapy to 53%, and neoadjuvant therapy to 10%. Regarding local liver-directed treatments, 18% underwent interventional procedures, radiotherapy was applied in 4%, and only two patients (0.23%) received high-intensity focused ultrasound (HIFU). Among patients receiving TCM, 72% were treated for less than 90 days. The cumulative mortality rates at 36 and 60 months were 84.0% (723/861) and 91.2% (785/861), respectively.

### 3.2 Univariate and multivariate Cox regression results

In univariate Cox regression analyses, age, interval from primary tumor to liver metastasis, hypertension, coronary heart disease, TNM stage, number of liver metastases, liver resection, chemotherapy, liver-directed intervention, immunotherapy, targeted therapy, neoadjuvant chemotherapy, and TCM doses were each significantly associated with OS in patients with CRLM.

**Figure 2:**
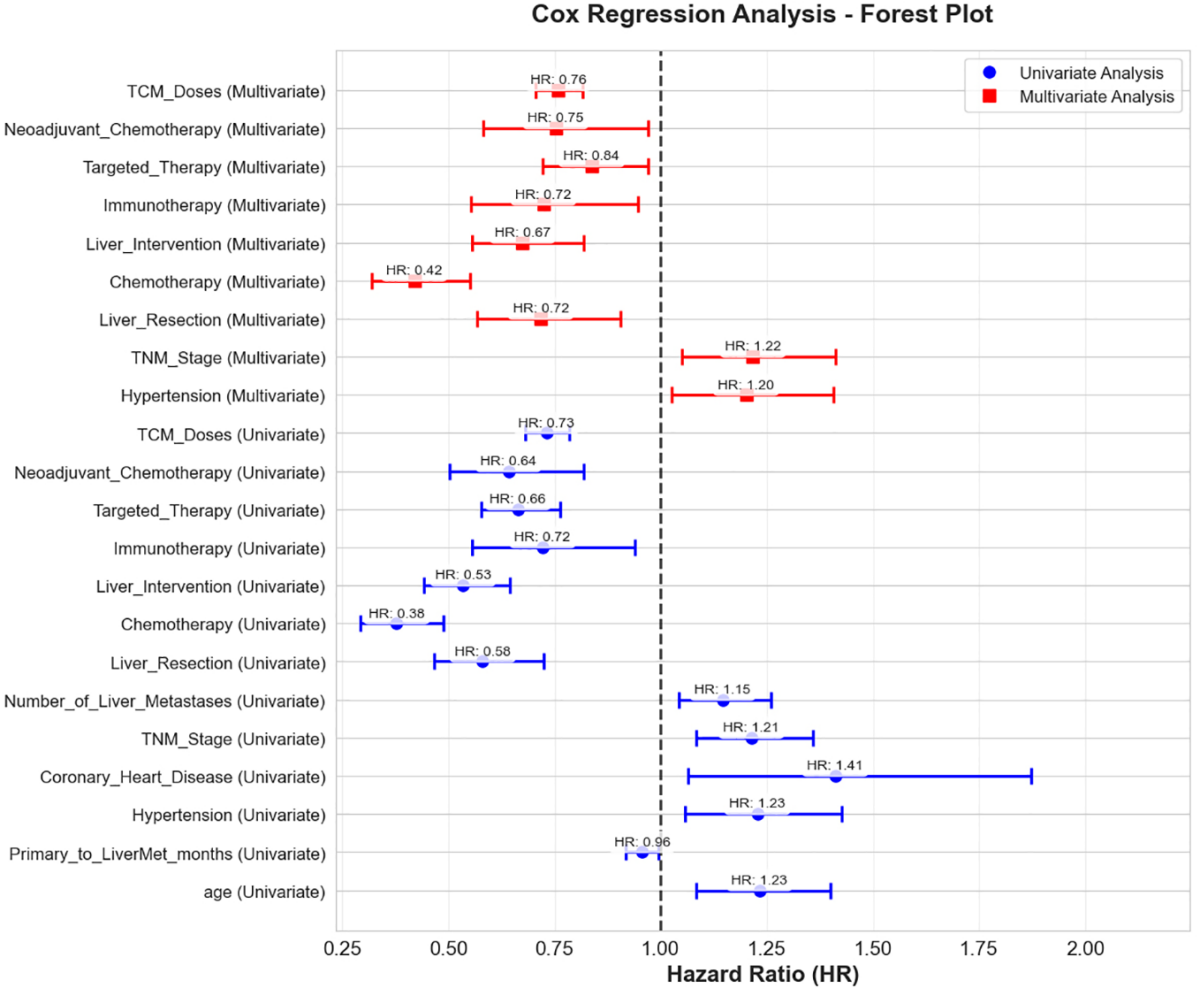
Cox regression analysis-forest plot. Blue circles represent hazard ratios (HRs) from univariate Cox regression, and red squares represent HRs from multivariate Cox regression. Horizontal lines indicate 95% CIs, and the vertical dashed line represents the reference line of no effect (HR = 1.0).

To adjust for potential confounding factors, multivariate Cox regression analyses were subsequently performed. The results indicated that hypertension, TNM stage, liver resection, chemotherapy, liver-directed intervention, immunotherapy, targeted therapy, neoadjuvant chemotherapy, and TCM doses were independent prognostic factors for OS (**Figure 2**; full results are provided in **Supplementary Table 1**).

### 3.3 Model development and comparison

Variables identified as independent prognostic factors in the multivariate Cox regression—including hypertension, TNM stage, liver resection, chemotherapy, liver-directed intervention, immunotherapy, targeted therapy, neoadjuvant chemotherapy, and TCM doses—were subsequently used as input features in machine learning models to predict 36-month and 60-month survival in patients with CRLM.

**Figure 3:**
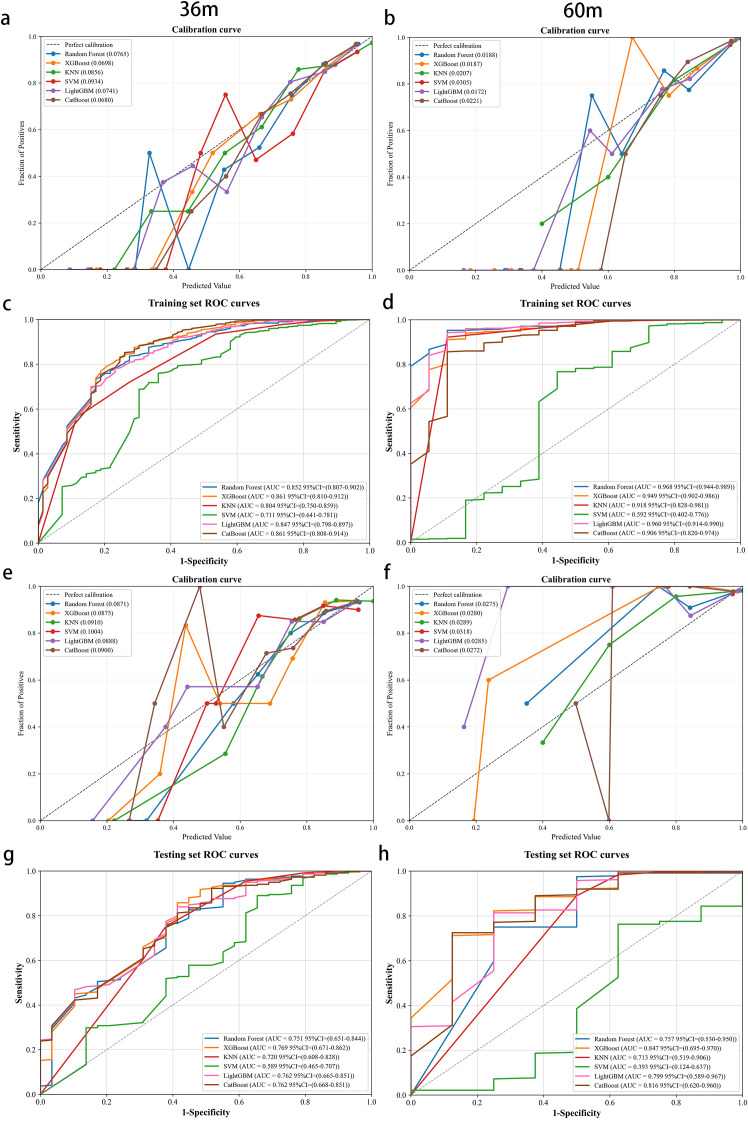
Performance evaluation of machine learning models for predicting 36- and 60-month survival in patients with CRLM. **(a, b)** Calibration curves in the training set, showing the agreement between predicted probabilities and observed outcomes for 36 months **(a)** and 60 months **(b)**. **(c, d)** ROC curves in the training set, illustrating the discriminative performance of each model. **(e, f)** Calibration curves in the testing set, assessing the generalizability of the models on an independent cohort. **(g, h)** ROC curves in the testing set, with XGBoost showing the highest AUC values for 36 months **(g)** and 60 months **(h)**.

**Figure 3** presents the ROC curves of different models in the training and testing sets, with corresponding area under the curve (AUC) values detailed in **Table 2**. For 36-month survival prediction, the XGBoost model demonstrated the best discriminative performance (testing set AUC = 0.769; training set AUC = 0.861), outperforming the other five models. At 60 months, overall model performance improved, with XGBoost still achieving the highest discrimination (testing set AUC = 0.847; training set AUC = 0.996). Calibration curves in the testing set further indicated that the XGBoost model exhibited good agreement between predicted probabilities and observed outcomes.

**Table 2:** Comparison of AUC performance of six machine learning models for predicting 36- and 60-month survival in patients with CRLM.

Model	36-month AUC [95%CI]	60-month AUC [95%CI]
Training set		
Random Forest	0.852 [0.807-0.902]	0.968 [0.944-0.989]
XGBoost	0.861 [0.810-0.912]	0.949 [0.902-0.986]
KNN	0.804 [0.750-0.859]	0.918 [0.828-0.981]
SVM	0.711 [0.641-0.781]	0.592 [0.402-0.776]
LightGBM	0.847 [0.798-0.897]	0.960 [0.914-0.990]
CatBoost	0.861 [0.808-0.914]	0.906 [0.820-0.974]
Testing set		
Random Forest	0.751 [0.651-0.844]	0.757 [0.530-0.950]
XGBoost	0.769 [0.671-0.862]	0.847 [0.695-0.970]
KNN	0.720 [0.608-0.828]	0.713 [0.519-0.906]
SVM	0.589 [0.465-0.707]	0.393 [0.124-0.637]
LightGBM	0.762 [0.665-0.851]	0.799 [0.589-0.967]
CatBoost	0.762 [0.668-0.851]	0.816 [0.620-0.960]

Confusion matrices illustrating the classification performance of each model for patient survival outcomes are presented in **Supplementary Figure 2**. At 36 months, XGBoost maintained high specificity while demonstrating acceptable sensitivity. With the extended 60-month prediction horizon, classification performance differences became more pronounced, with XGBoost providing the most balanced prediction of both death (class 0) and survival (class 1). In contrast, models such as SVM and Random Forest showed limitations when handling class imbalance, bias toward predicting survival. These findings further confirm the robustness of XGBoost, justifying its selection as the final predictive model for subsequent analyses. The XGBoost model was trained using the following hyperparameters: n_estimators = 200, max_depth = 3, learning_rate = 0.01, subsample = 1.0, and colsample_bytree = 1.0. Hyperparameters not explicitly specified were retained at their default settings in XGBoost version 2.0.3.

### 3.4 SHAP analysis reveals key predictors in the 36-month survival model

To enhance model interpretability, SHAP analysis was performed to the 36- and 60-month survival prediction models, quantifying the contribution of each feature to model outputs at three levels: global importance, local effect, and individual prediction.

**Figure 4:**
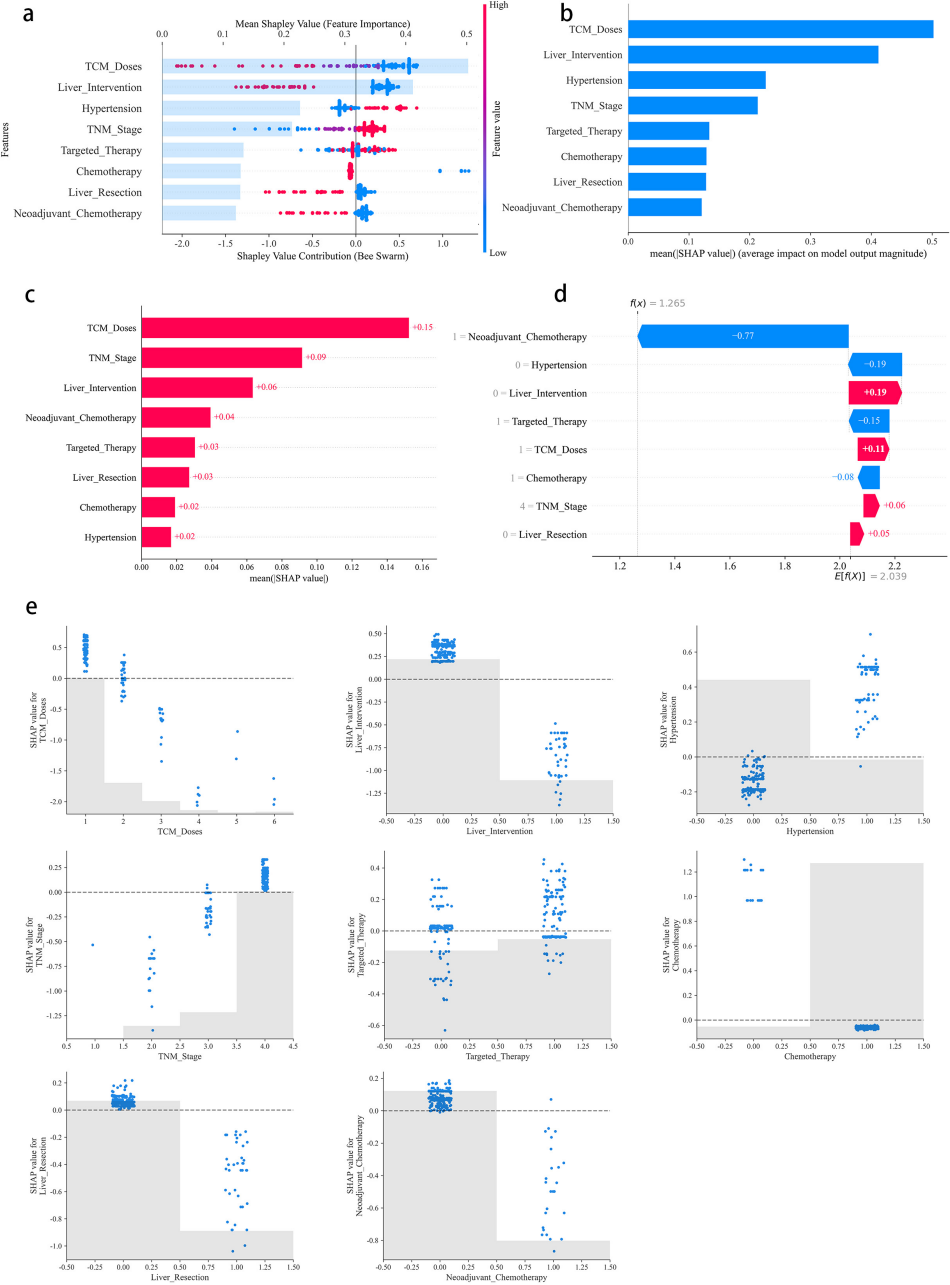
SHAP-based interpretability analysis of the 36-month survival prediction model. **(a)** SHAP beeswarm plot distribution of SHAP values across the cohort. Features are ranked by mean absolute SHAP value. Each dot represents an individual sample. Color represents feature value (red: high; blue: low). **(b)** Bar plot of mean absolute SHAP values demonstrating the global importance ranking of each feature. **(c)** Alternative visualization of feature importance based on mean |SHAP| values. **(d)** SHAP waterfall plot for a representative patient, illustrating how individual features cumulatively contribute to the final predicted outcome relative to the baseline value. **(e)** SHAP dependence plots of key variables, showing the relationship between feature values and their corresponding SHAP contributions.

**Figure 4A** presents the SHAP beeswarm plot for the 36-month model. Traditional Chinese medicine dose (TCM_Doses) showed the highest mean absolute SHAP value, indicating it was the most important predictor. The next most important features were liver-directed intervention (Liver_Intervention), hypertension, TNM stage (TNM_Stage), targeted therapy (Targeted_Therapy), chemotherapy, liver resection (Liver_Resection), and neoadjuvant chemotherapy (Neoadjuvant_Chemotherapy).

The beeswarm plot further demonstrated consistent directional effects of key variables. Higher TCM_Doses values were associated with negative SHAP values, suggesting a reduced predicted risk with increasing TCM dosage. In contrast, higher TNM stage values were associated with positive SHAP values, indicating increased risk in advanced disease. Liver intervention (value = 1) was predominantly associated with negative SHAP values, suggesting a protective effect. Hypertension contributed positively to SHAP values, indicating increased mortality risk. Patients receiving targeted therapy or neoadjuvant chemotherapy generally showed negative SHAP contributions, consistent with improved predicted survival.

Quantitative ranking based on mean absolute SHAP values (mean |SHAP|, **Figures 4B, C**) further confirmed these findings. TCM dose had a mean |SHAP| of approximately 0.15, notably higher than TNM stage (approximately 0.09) and liver-directed intervention (approximately 0.06), with other variables contributing progressively less. These results indicate that the predictive contribution of TCM dosage in 36-month survival estimation exceeds that of traditional TNM staging.

**Figure 4D** shows a SHAP waterfall plot for an individual patient. In this case, Neoadjuvant_Chemotherapy, Hypertension, and Liver_Intervention had negative SHAP values (blue), reducing the predicted risk, whereas TCM_Doses, TNM_Stage, and Liver_Resection contributed positive SHAP values (red). The cumulative impact of these features highlights the model’s ability to individualize prediction patterns.

**Figure 4E** illustrates SHAP dependence plots for several key features, demonstrating how changes in feature values influence model predictions. Notably, TCM_Doses exhibited a clear dose–response relationship, with higher doses associated with lower SHAP values, potentially indicating a protective effect or reduced risk at elevated doses. Liver_Intervention and Hypertension showed distinct SHAP value distributions based on their status, reflecting the substantial impact of clinical interventions and comorbidities on patient prognosis. Additionally, SHAP distributions for TNM_Stage, Targeted_Therapy, Chemotherapy, Liver_Resection, and Neoadjuvant_Chemotherapy revealed a high degree of heterogeneity, underscoring the context-dependent nature of these clinical determinants on model outputs.

### 3.5 SHAP analysis reveals key predictors in the 60-month survival model

**Figure 5:**
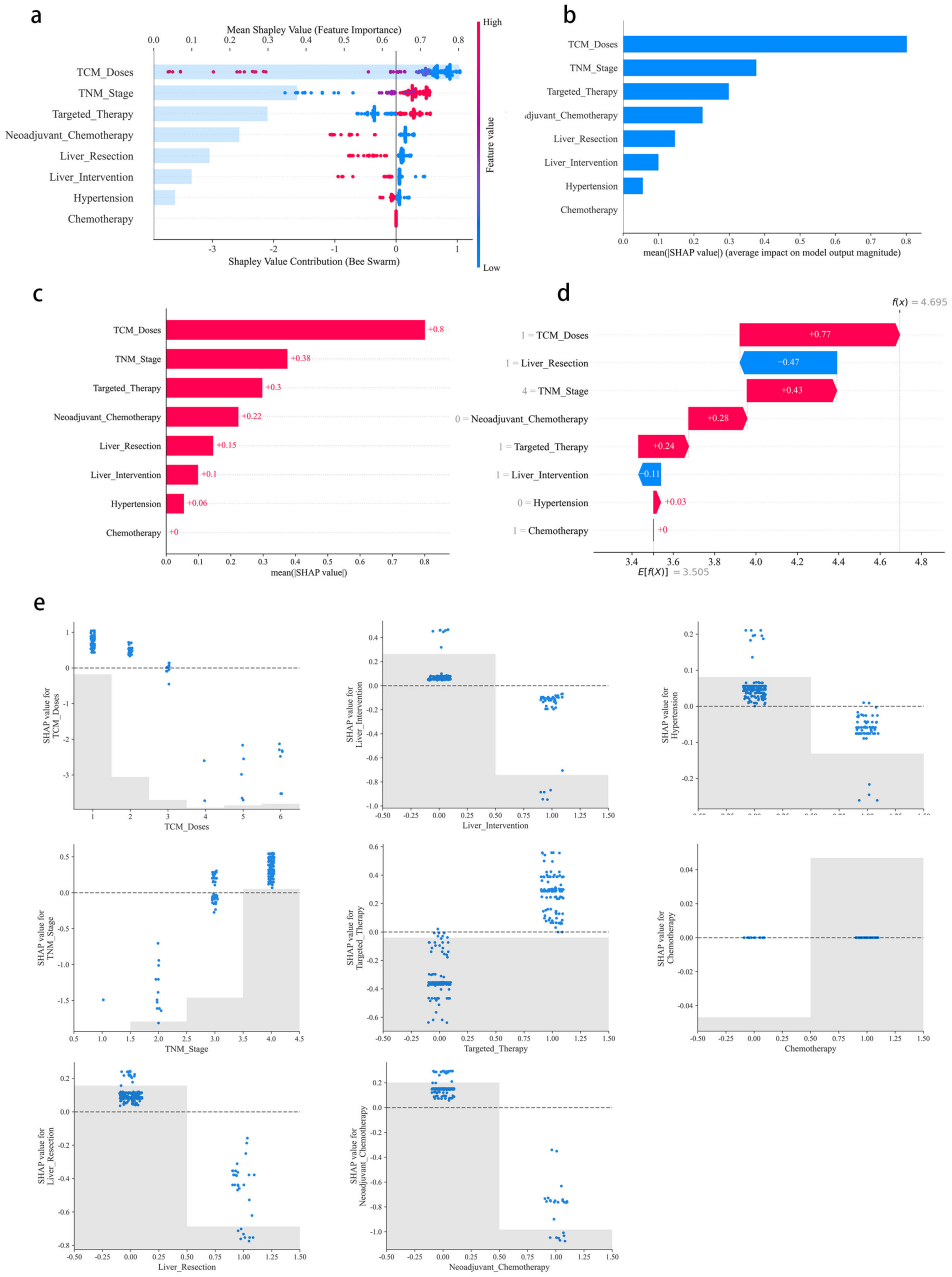
SHAP-based interpretability analysis of the 60-month survival prediction model. The panels correspond to the same analyses as shown in **Figure 4**, including the SHAP beeswarm plot **(a)**, global feature importance bar plot **(b)**, alternative feature importance visualization **(c)**, SHAP waterfall plot for a representative patient **(d)**, and SHAP dependence plots of key variables **(e)**.

To evaluate robustness of the model and consistency of feature contributions in longer-term survival prediction, SHAP analysis was also performed for the 60-month survival prediction model (**Figure 5**).

The overall patterns were consistent with the 36-month model. Traditional Chinese medicine dose (TCM_Doses) remained the most influential predictor, with a markedly higher mean |SHAP| value (≈0.80), indicating an even greater contribution to prediction in long-term survival assessment. TNM stage (TNM_Stage) remained the second most important feature, underscoring tumor staging as a stable and core risk factor across different time horizons.

Notably, cross-model comparisons revealed distinct dynamic shifts in feature importance when extending the prognostic horizon beyond 36 months. Specifically, the predictive weight of TCM_Doses became substantially more pronounced, suggesting that TCM interventions may exert sustained, cumulative benefits on long-term survival. Furthermore, the contributions of Targeted_Therapy and Neoadjuvant_Chemotherapy increased, reflecting the continued impact of systemic treatments on long-term outcomes; the overall SHAP distribution for chemotherapy approached zero, indicating a relatively limited independent effect on long-term predictions; liver resection and liver-directed interventions continued to act as protective factors, though their relative importance was slightly lower than in the 36-month model.

Dependence plots further revealed that TCM_Doses maintained a clear dose–response relationship in the 60-month model, with an wider distribution of SHAP values range, TCM_Doses exhibited stronger discriminatory ability.

### 3.6 Kaplan–Meier survival analysis stratified by TCM_doses

**Figure 6:**
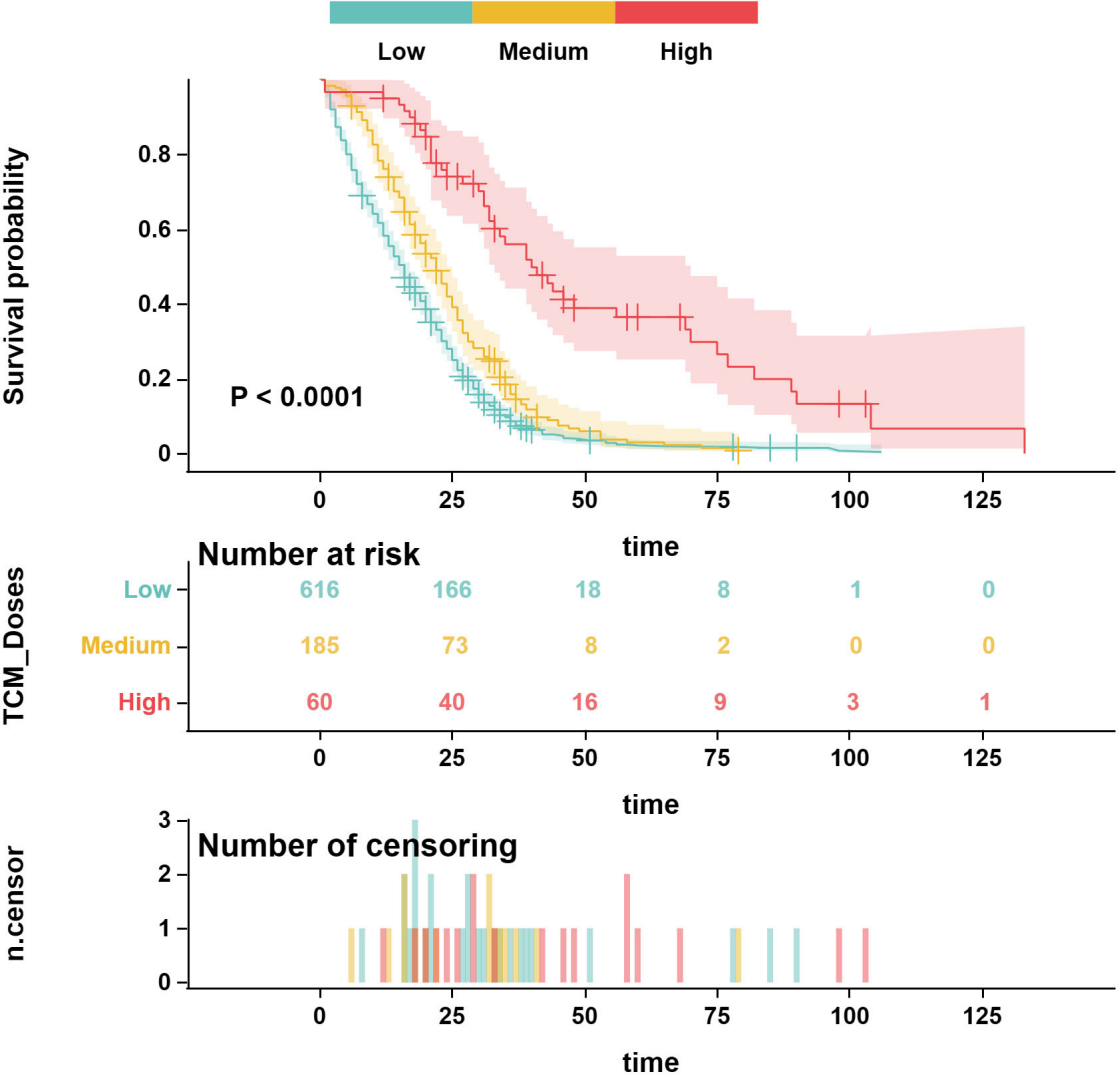
Kaplan–Meier survival analysis according to TCM doses levels. Patients were classified into three distinct cohorts (low-, medium-, and high-dose) based on their clinical TCM exposure.categorize Shaded areas represent 95% CIs. The number at risk and the number of censoring events over time are shown below the survival curves. Statistical significance was assessed using the log-rank test.

Given that SHAP analysis identified TCM_Doses as a pivotal predictor within our prognostic framework, we further evaluated its clinical risk-stratification capacity using long-term follow-up data. Patients were stratified into three distinct cohorts based on their exposure levels (Low, Medium, and High TCM_Doses). Subsequently,Kaplan–Meier survival analysis was performed to estimate cumulative survival probabilities over time (**Figure 6**).

The Kaplan–Meier curves demonstrated clear separation among the three groups, with stratification trends persisting over time. At risk, the low, medium, and high dosage cohorts comprising 616, 185, and 60 patients, at risk at baseline, respectively. Log-rank (Mantel–Cox) testing indicated highly significant differences between groups (*χ*² = 83.260, *P* < 0.001; **Supplementary Table 2**). Pairwise comparisons confirmed statistically significant survival differences between all groups (**Supplementary Table 2**). Analysis of survival times revealed a stepwise prolongation in median survival time corresponding to higher TCM_Doses levels. Notably, the median survival in the high-dosage cohort was approximately 2.5 -fold that of the low-dosage cohort, indicating a clear dose–response relationship (**Supplementary Table 2**).

Overall the Kaplan–Meier analysis was consistent with SHAP-derived feature attribution. TCM_Doses demonstrated not only high importance in the machine learning model but also showed strong risk stratification ability in traditional survival analysis, further supporting its key prognostic value in patients with CRLM.

### 3.7 Temporal validation

To further evaluate the robustness of our model, clinical and outcome data were collected from 196 CRLM patients treated at our center from January to December 2024. The discriminative performance, calibration, and clinical utility of the XGBoost model were systematically assessed.

**Figure 7:**
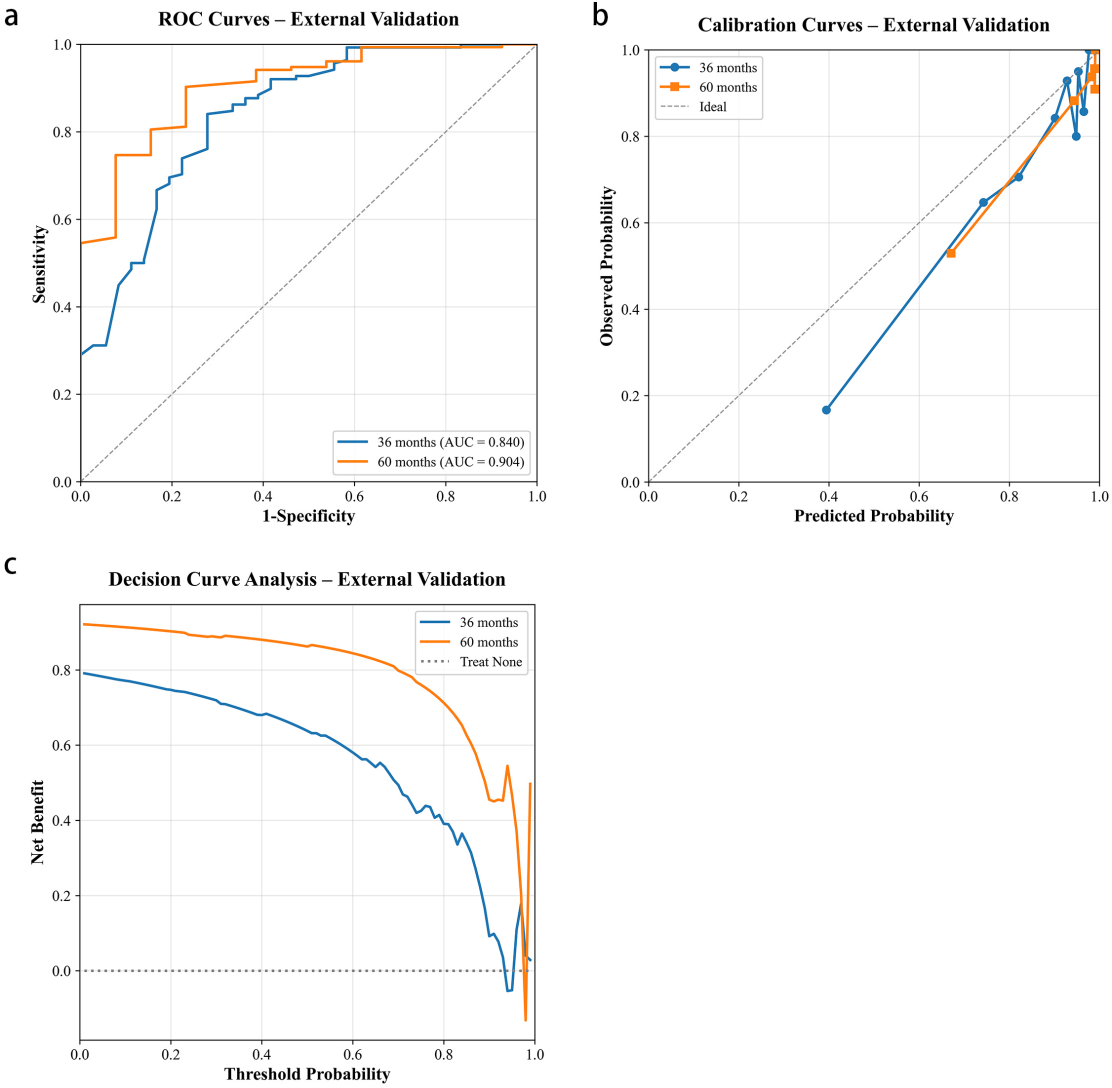
Temporal validation of the 36- and 60-month survival prediction models. **(a)** ROC curves for 36- and 60-month survival prediction. **(b)** Calibration plots demonstrating agreement between predicted and observed survival probabilities. **(c)** Decision curve analysis illustrating the clinical utility of the models.

As shown in **Figure 7A**, ROC curve analysis demonstrated discriminatory performance for both 36- and 60-month survival predictions, with AUCs of 0.840 and 0.904, respectively. Calibration curves (**Figure 7B**) showed good agreement between predicted survival probabilities and observed outcomes in the temporal cohort, indicating satisfactory calibration. Decision curve analysis (**Figure 7C**) demonstrated that the model provided higher net benefit than the “treat-none” strategy across a wide range of threshold probabilities for both time horizons, with the 60-month model showing greater net benefit over most thresholds.

Collectively, the temporal validation results indicate that the model maintains stable discriminative performance, good calibration, and potential clinical applicability in an independent dataset.

### 3.8 Clinical application

**Figure 8:**
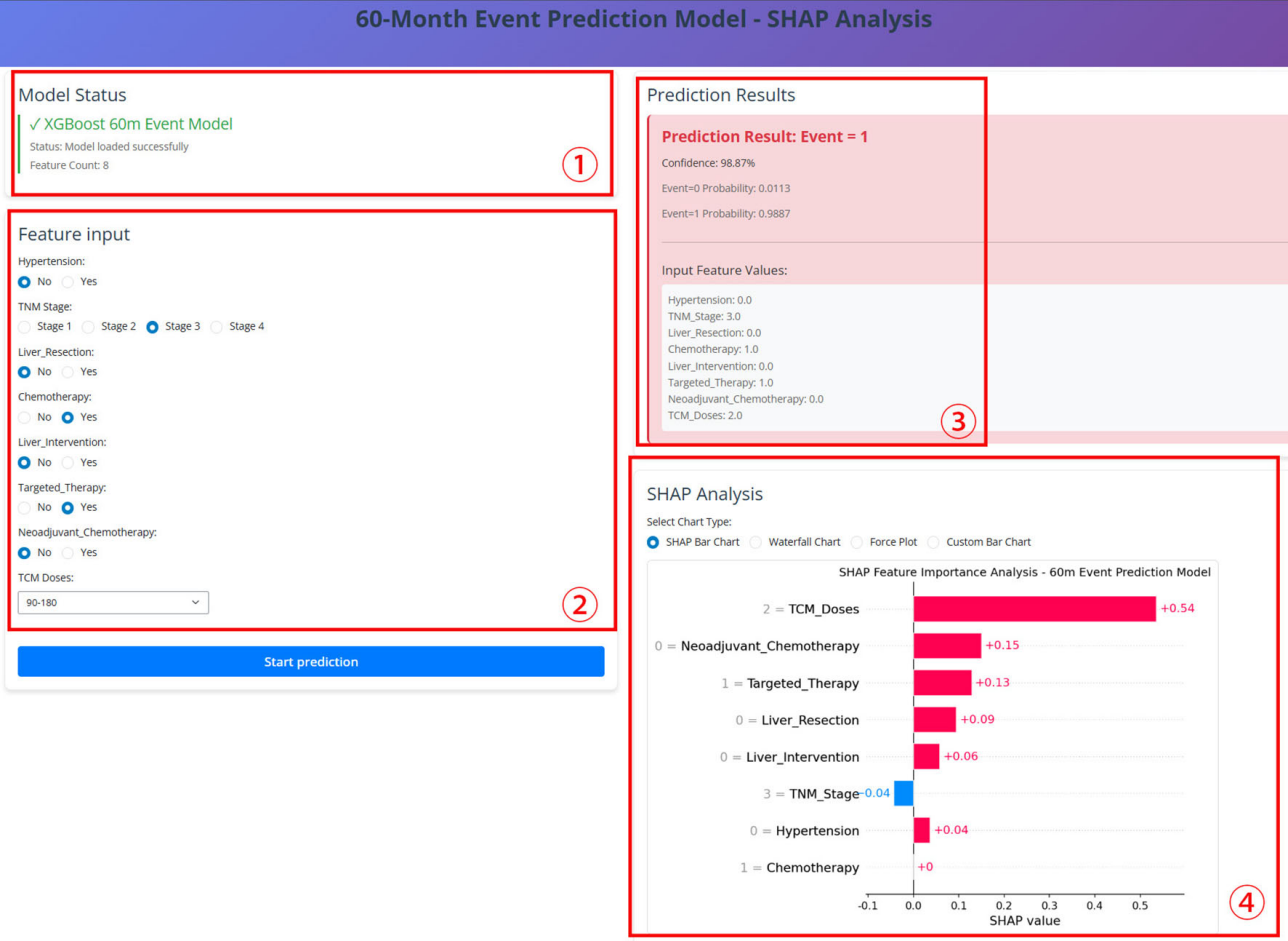
Web interface of the clinical prediction platform. Operation steps: Step 1: Confirm that the model has successfully loaded in the “Model Status” section. Step 2: Enter the patient’s eight clinical variables in the “Feature Input” section. Step 3: Click the “Start Prediction” button to generate the predicted probability of a 60-month event. Step 4: Select the desired SHAP visualization type in the “SHAP Analysis” section to explore feature contributions.

To facilitate translational utility and seamless clinical decision-making, an interactive, web-based application was developed and publicly deployed utilizing the R Shiny framework (available at: https://pumpkin227.shinyapps.io/apps_1_15/)(**Figure 8**). This digital platform fully encapsulates our core 60-month prognostic model constructed via the XGBoost algorithm. By inputting patient-specific variables, clinicians can intuitively generate individualized long-term survival probability estimations and risk stratifications for patients diagnosed with CRLM, thereby bridging the gap between complex machine learning and bedside application.

The model integrates eight clinical variables—Hypertension, TNM Stage, Liver Resection, Chemotherapy, Liver Intervention, Targeted Therapy, Neoadjuvant Chemotherapy, and TCM Doses—and provides individualized risk predictions based on patient-specific clinical inputs. To enhance interpretability, a SHAP-based explanation module was incorporated, enabling multiple visualization outputs, including waterfall charts, force plots, and bar charts, which quantify the contribution of each feature to individual predictions.

This platform enables individualized prognostic estimation, identification of key influencing factors, and graphical feature attribution, thereby supporting clinical risk stratification and decision-making. By pairing real-time algorithmic predictions with transparent, verifiable feature contributions, the system effectively demystifies the “black box” nature of machine learning, substantially augmenting the model’s interpretability, clinical intuition, and translational viability.

## 4 Discussion

In this study, we developed and validated an interpretable machine learning model to predict 60-month mortality risk in patients with CRLM using a retrospective cohort of 861 patients. Through comparison across multiple algorithms, XGBoost demonstrated the highest discriminative performance for both 36- and 60-month survival predictions, and demonstrated robust performance in discrimination, calibration, and clinical utility in a temporal validation cohort. SVM and KNN demonstrated inferior performance for several reasons. KNN is highly sensitive to data distribution, feature scaling, and sample sparsity, and its predictive stability may decrease in relatively small or imbalanced datasets. Similarly, SVM performance is strongly influenced by kernel selection and parameter tuning, and it may not adequately capture complex nonlinear relationships when the underlying data structure is heterogeneous. By integrating SHAP analysis with Kaplan–Meier survival assessment, this study interpreted contribution of individual clinical variables to survival outcomes and highlighted the significant role of TCM dosage in predicting medium- to long-term prognosis.

The clinical prognosis of patients with CRLM is characterized by profound heterogeneity. While traditional Cox regression models offer interpretability, they are limited in capturing nonlinear relationships and complex interactions among variables. In this study, we employed the XGBoost algorithm, achieving AUCs of 0.769 and 0.847 for 36- and 60-month survival predictions in the test set, respectively, demonstrating enhanced discriminative power over longer prognostic horizons. Notably, in atemporal validation cohort, the 60-month AUC reached 0.904, indicating strong generalizability of the model for long-term prediction.

Calibration curves demonstrated excellent agreement between predicted probabilities and observed outcomes, and decision curve analysis further confirmed higher net benefit across a wide range of threshold probabilities. This multidimensional evaluation—from discrimination and calibration to clinical utility—supports the predictive performance.

SHAP analysis indicated that TNM stage consistently ranked among the top features in both the 36- and 60-month models, highlighting its role as a key prognostic factor reflecting tumor burden and disease stage in long-term survival. Liver resection and liver-directed interventions acted as protective factors at both time points, aligning with prior clinical evidence that local therapies can confer survival benefits in selected patients.

The contributions of targeted therapy and neoadjuvant chemotherapy increased in long-term predictions, suggesting that systemic treatments exert a sustained impact on extended prognosis. Notably, chemotherapy exhibited a near-zero SHAP distribution in the 60-month model, indicating that its independent long-term contribution may be influenced by other treatments and potential patient selection biases. This temporal variation in feature importance represents a dynamic characteristic that is difficult to capture intuitively with conventional Cox models.

One notable finding of this study is that TCM_Doses consistently ranked as the most important predictor in both the 36- and 60-month models. In particular, the 60-month model showed a marked increase in the mean |SHAP| value for TCM_Doses, far exceeding that of other clinical factors, indicating a contribution to prediction of TCM_Doses had stronger influence in the prediction model. SHAP beeswarm and dependence plots revealed a clear dose–response relationship, with higher TCM doses corresponding to lower SHAP values, suggesting a trend toward reduced risk. This nonlinear dose–response pattern was effectively characterized by the machine learning model.

Further Kaplan–Meier estimations validated a stepwise survival advantage favoring higher TCM_Doses, supported by highly significant log-rank statistics. Notably, the median survival time in the High-dose group was approximately 2.5 times that of the low-dose cohort. This alignment between traditional epidemiological survival tracking and machine-learning interpretability forms a robust cross-methodological verification loop. Although the observational nature of this cohort precludes the establishment of direct causality, these parallel findings indicate that TCM maintenance could offer sustained and cumulative benefits for long-term CRLM management, underscoring the necessity for future prospective investigation.

The “black-box” nature of machine learning models remains a major obstacle to their clinical implementation. In this study, SHAP-based interpretability was incorporated at three levels, including global feature importance, directionality of variable effects, and individual risk contributions, thereby enhancing transparency of the prediction process. For instance, individual-level waterfall plots clearly illustrate the cumulative contributions associated with increased or decreased predicted risk of different features. Such personalized risk attribution aids clinicians in identifying key drivers of high risk and may assist clinical decision-making. By focusing not only on predictive performance metrics, such as AUC, but also on why a prediction is made, this approach enhances the clinical interpretability of the model.

The model was further validated in an temporal validation cohort of 196 patients, achieving a 60-month AUC of 0.904, with calibration and decision curve analyses demonstrating robust predictive performance. These results further support for the model’s generalizability. While many predictive models are limited to internal validation, our temporal validation indicates that the model maintains stable performance across temporally separated cohorts, supporting its potential for broader clinical application.

Based on the final XGBoost model, an open-access web application was established to provide real-time risk scoring and SHAP-derived interpretability. Compared with traditional static nomograms, this platform offers dynamic updates and individualized explanations, facilitating risk stratification, tailoring of follow-up intensity, and enhanced clinician–patient communication. This dual design of “real-time prediction and explainable attribution” effectively bridges the gap between abstract mathematical modeling and practical clinical decision support systems, delivering a tangible solution for precision medicine deployment.

Several limitations should be acknowledged. First, as a single-center retrospective study, selection bias cannot be excluded. Second, although temporal validation was performed, the validation cohort size was relatively limited, and further multicenter studies with larger cohorts are warranted. Third, the quantification of TCM_Doses did not account for specific formulas or herbal composition; future studies integrating detailed formulation data and underlying molecular mechanisms may provide more refined analyses. Fourth, molecular profiling information, such as RAS/BRAF mutations or MSI status, was not included in this study. Integrating molecular characteristics with dynamic treatment variables may further optimize model performance in subsequent investigations. Fifth, although XGBoost-based models performed well, they are not dedicated survival models, and more advanced approaches such as XGBSE may further improve survival function estimation and uncertainty quantification. Sixth, SHAP-based feature importance reflects model-derived associations rather than causal effects; in particular, the high importance of chemotherapy may be influenced by its high prevalence in the cohort (approximately 92%), introducing potential imbalance-related bias. Finally, the proposed web-based prediction tool has not undergone formal usability testing or prospective clinical validation and is not yet integrated with electronic medical record systems, which may limit its immediate clinical applicability. Future studies should therefore focus on prospective multicenter validation, standardized harmonization of TCM dose assessment, and real-world implementation evaluation, including usability testing and clinical decision-support integration.

In summary, this study applied an interpretable machine learning framework to identify key determinants of medium- and long-term survival in CRLM and systematically evaluated the role of TCM intervention dosage in survival prediction. Through temporal validation and deployment of an online platform, we provide a tool with potential clinical utility for individualized risk assessment and therapeutic decision support in CRLM. Future studies should focus on prospective multicenter validation and further model refinement by integrating molecular features and longitudinal treatment data to improve predictive performance.

## Data Availability

The datasets generated and analyzed during the current study are not publicly available due to patient privacy and institutional restrictions but are available from the corresponding author on reasonable request and with permission from the relevant ethics committee.
