# Supplementary material for: Survival prediction in colorectal cancer liver metastases using machine learning with SHAP-based interpretation
Source: Front Oncol. 2026 Jun 10;16:1836991. doi: 10.3389/fonc.2026.1836991 (PMC13290457; doi:10.3389/fonc.2026.1836991)
Supplement: Supplementary file 1 [file DataSheet1.docx]

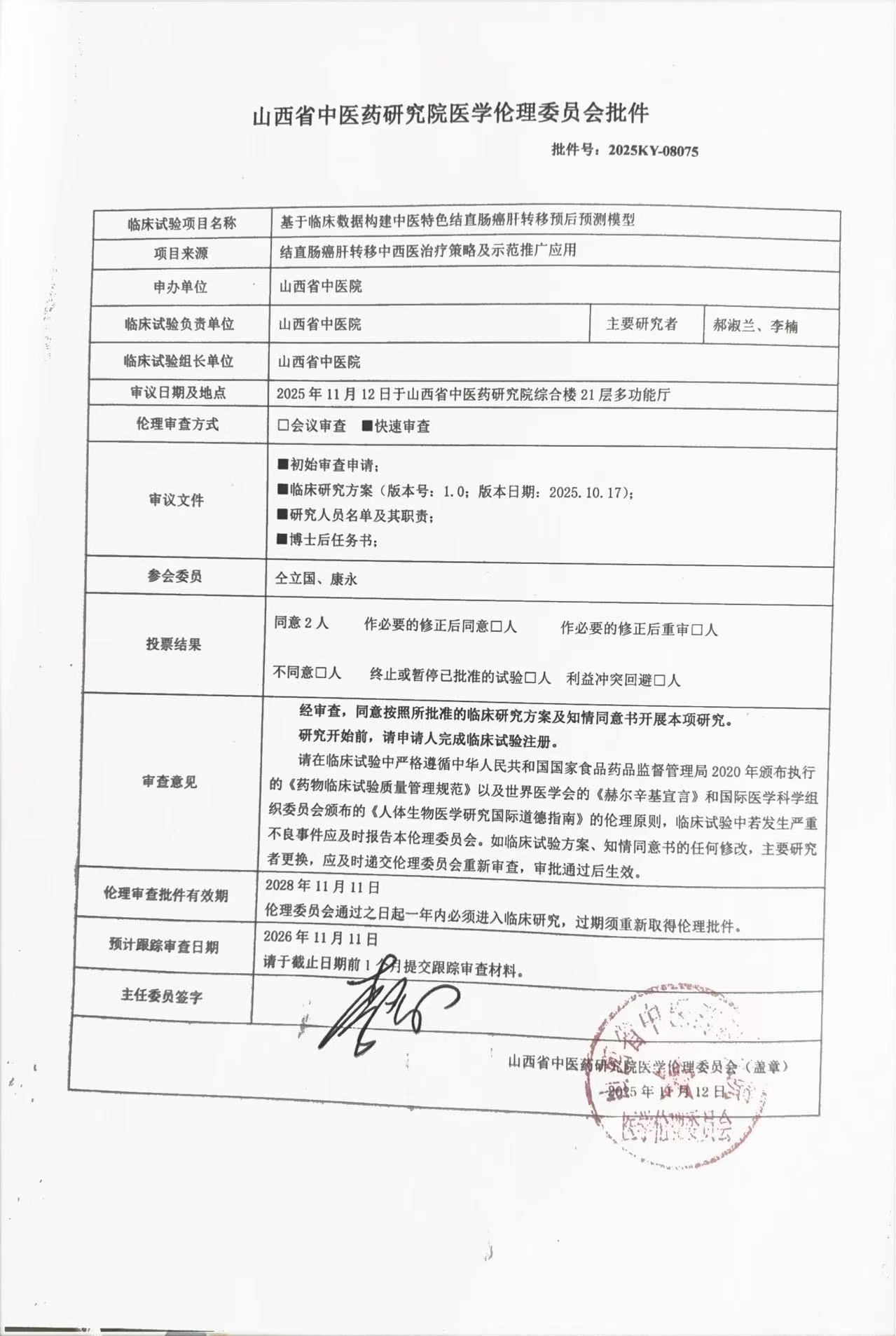


**Supplementary Fig S1. Ethics Approval Document**


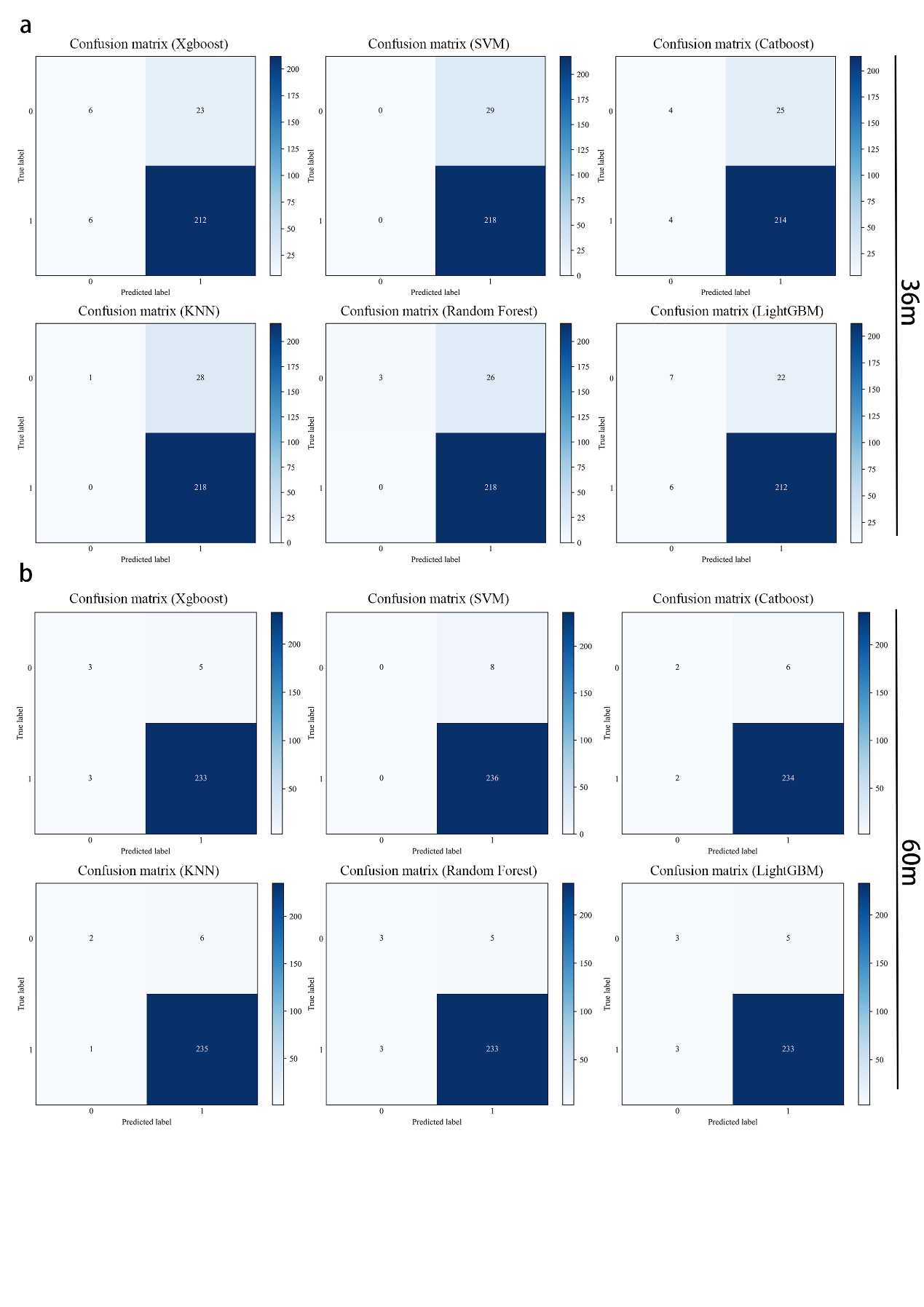


**Supplementary Fig S2. Confusion matrices of different machine learning models for 36- and 60-month survival prediction.**

Note: The top two rows depict the 36-month survival predictions, while the bottom two rows show the 60-month predictions. The horizontal axis (Predicted Label) represents the survival status predicted by the model (0 = death, 1 = survival), and the vertical axis (True Label) represents the observed survival status (0 = death, 1 = survival). Color intensity corresponds to the number of samples, with darker shades indicating larger sample sizes. Values along the diagonal—top-left for true negatives and bottom-right for true positives—reflect the model’s ability to correctly classify outcomes.

**Supplementary Table S1. Univariate and Multivariate Cox Regression Analyses of Prognos**tic Factors

| **Feature** | **HR** | **95 CI** | ***P*_Value** | **Significant** |
| --- | --- | --- | --- | --- |
| **Single factor** |  |  |  |  |
| Age | 1.23 | [1.085-1.401] | 0.001 | ** |
| Primary_to_LiverMet_months | 0.96 | [0.918-0.996] | 0.030 | * |
| Sex | 0.97 | [0.845-1.12] | 0.698 | ns |
| Hypertension | 1.23 | [1.057-1.425] | 0.007 | ** |
| Diabetes | 1.01 | [0.826-1.238] | 0.914 | ns |
| Coronary_Heart_Disease | 1.41 | [1.064-1.871] | 0.017 | * |
| Stroke | 1.13 | [0.825-1.552] | 0.443 | ns |
| Hepatitis_B | 1.63 | [0.844-3.155] | 0.145 | ns |
| Primary_Site | 1.02 | [0.992-1.048] | 0.161 | ns |
| TNM_Stage | 1.21 | [1.083-1.359] | 0.001 | *** |
| Histology_Primary | 1.01 | [0.912-1.129] | 0.794 | ns |
| Number_of_Liver_Metastases | 1.15 | [1.043-1.259] | 0.005 | ** |
| Extrahepatic_Metastasis | 1.05 | [0.911-1.203] | 0.521 | ns |
| Liver_Resection | 0.58 | [0.466-0.724] | 0.000 | *** |
| Chemotherapy | 0.38 | [0.293-0.489] | 0.000 | *** |
| Liver_Radiotherapy | 0.77 | [0.536-1.112] | 0.165 | ns |
| Liver_Intervention | 0.53 | [0.442-0.645] | 0.000 | *** |
| Liver_HIFU | 1.67 | [0.417-6.7] | 0.469 | ns |
| Immunotherapy | 0.72 | [0.556-0.938] | 0.015 | * |
| Targeted_Therapy | 0.66 | [0.577-0.763] | 0.000 | *** |
| Neoadjuvant_Chemotherapy | 0.64 | [0.503-0.818] | 0.000 | *** |
| TCM_Doses | 0.73 | [0.681-0.784] | 0.000 | *** |
| **Multiple factors** |  |  |  |  |
| Age | 1.04 | [0.909-1.197] | 0.548 | ns |
| Primary_to_LiverMet_months | 1.02 | [0.967-1.072] | 0.500 | ns |
| Hypertension | 1.20 | [1.025-1.407] | 0.023 | * |
| Coronary_Heart_Disease | 1.06 | [0.785-1.43] | 0.705 | ns |
| TNM_Stage | 1.22 | [1.049-1.412] | 0.010 | ** |
| Number_of_Liver_Metastases | 1.07 | [0.974-1.181] | 0.154 | ns |
| Liver_Resection | 0.72 | [0.568-0.905] | 0.005 | ** |
| Chemotherapy | 0.42 | [0.32-0.55] | 0.000 | *** |
| Liver_Intervention | 0.67 | [0.556-0.819] | 0.000 | *** |
| Immunotherapy | 0.72 | [0.554-0.946] | 0.018 | * |
| Targeted_Therapy | 0.84 | [0.721-0.971] | 0.019 | * |
| Neoadjuvant_Chemotherapy | 0.75 | [0.583-0.971] | 0.029 | * |
| TCM_Doses | 0.76 | [0.705-0.816] | 0.000 | *** |

**Table S2. Kaplan–Meier survival analysis according to TCM_Doses levels**

**(1) Overall comparison of survival curves (Log-rank test)**

| **Test** | **χ²** | **df** | ***P* value** |
| --- | --- | --- | --- |
| Log-rank (Mantel–Cox) | 83.260 | 2 | <0.001 |

**(2) Pairwise comparisons between groups (Log-rank test)**

| **Comparison** | **χ²** | ***P* value** |
| --- | --- | --- |
| Low vs. Medium | 16.469 | <0.001 |
| Low vs. High | 69.252 | <0.001 |
| Medium vs. High | 41.904 | <0.001 |

**(3) Survival time estimates by TCM_Doses level**

| **Group** | **Mean survival time (95% CI)** | **SE** | **Median survival time**  **(95% CI)** | **SE** |
| --- | --- | --- | --- | --- |
| Low | 18.555 (17.244–19.866) | 0.669 | 16.000 (14.555–17.445) | 0.737 |
| Medium | 24.089 (21.863–26.315) | 1.136 | 22.000 (19.445–24.555) | 1.304 |
| High | 52.760 (42.466–63.054) | 5.252 | 40.000 (30.295–49.705) | 4.951 |
| Overall | 22.090 (20.688–23.491) | 0.715 | 18.000 (16.639–19.361) | 0.694 |
